# Soil Moisture and Vapor Pressure Deficit Affect Ecosystem Water Use Efficiency via Modulating Gross Primary Productivity to Transpiration Ratio in Rainfed Maize in Northeast China

**DOI:** 10.3390/plants15081190

**Published:** 2026-04-13

**Authors:** Yangjie Guo, Zijun Zhu, Yuheng Zhang, Weinan Yao, Zhixian Li, Yuping Lv

**Affiliations:** College of Hydraulic Science and Engineering, Yangzhou University, Yangzhou 225009, China; 18452079057@163.com (Y.G.); 2401094279yjq@gmail.com (Z.Z.); 1748504141zyh@gmail.com (Y.Z.); 15151362718@163.com (W.Y.); 13405163085@163.com (Z.L.)

**Keywords:** evapotranspiration partitioning, underlying water use efficiency, carbon-water coupling, atmospheric aridity, soil moisture limitation

## Abstract

The distinct co-occurrence of soil water content (*SWC*) and vapor pressure deficit (*VPD*) influences ecosystem water use efficiency (*WUE*) by modifying the synergistic relationship between gross primary productivity (*GPP*) and evapotranspiration (*ET*), yet how they impact each other remains unclear in agricultural ecosystems. Based on long-term eddy covariance flux data (2005–2014) observed at a rainfed maize site in Northeast China, we examined how *SWC* and *VPD* affect *WUE* by decomposing it into gross primary productivity to transpiration ratio (*GPP*/*T*) and transpiration to evapotranspiration ratio (*T*/*ET*). Results showed that *WUE* was more sensitive to *VPD* than *SWC*. Increasing *VPD* directly suppressed *WUE* under all soil moisture conditions, whereas *SWC* had a context-dependent effect: higher *SWC* reduced *WUE* under low *VPD* but enhanced *WUE* under high *VPD*. The underlying mechanism was that changes in *GPP*/*T* (plant physiological regulation) dominated the *WUE* responses to both *SWC* and *VPD* (contributing 70.25–83.30% and 67.89–87.96%, respectively), while *T*/*ET* (evapotranspiration partitioning) played a minor role (<18%). Therefore, to improve *WUE* under future drier climates, agronomic practices should focus on enhancing photosynthetic capacity and stomatal regulation (e.g., selecting drought-tolerant varieties, optimizing nitrogen supply) rather than solely reducing soil evaporation. Furthermore, supplemental irrigation applied specifically during periods of high *VPD* (when atmospheric demand is strong) can effectively enhance *WUE*, as soil moisture becomes critically beneficial under such conditions. These findings provide a mechanistic basis for improving water use efficiency in rainfed maize systems under climate change.

## 1. Introduction

Water Use Efficiency (*WUE*), a key parameter characterizing the coupling relationship between ecosystem carbon and water, is defined as the ratio of gross primary productivity (*GPP*) to evapotranspiration (*ET*) (*WUE* = *GPP*/*ET*), quantifying carbon assimilation per unit of water loss via evapotranspiration [1,2]. Access to clean and sufficient water for use is of great importance for organic life in terrestrial systems under climate change [3,4]. In agricultural systems, *WUE* optimization is particularly significant for sustainable intensification, as it enables yield improvement without escalating water demand [5]. However, *WUE* regulation involves complex interactions among environmental drivers, with soil water content (*SWC*) and vapor pressure deficit (*VPD*) emerging as dominant factors through their divergent biophysical controls on *GPP*-*ET* equilibrium [6,7,8].

SWC primarily controls root-zone water availability by regulating plant hydraulic conductivity [9]. When moisture levels are low, stomatal closure reduces transpiration (*T*), which simultaneously limits RuBisCO activity and electron transport rates, leading to decreases in *GPP* and *E* [10,11]. Furthermore, *SWC* influences *WUE* indirectly by adjusting the transpiration to evapotranspiration ratio (*T/ET*)—a process affected by canopy structure and soil surface energy distribution [12,13]. *VPD*, reflecting atmospheric aridity, intensifies plant water stress as it increases, triggering stomatal closure to reduce transpiration, yet potentially inducing a “trade-off effect” between photosynthetic carbon assimilation and water loss [6,14]. Notably, the coupling between *SWC* and *VPD* exhibits spatiotemporal heterogeneity: their synergistic effects dominate at long-term scales, whereas their decoupling at short-term scales (e.g., hourly) enables quantification of their individual impacts [15,16]. Existing studies suggest that ecosystem carbon-water fluxes are generally more sensitive to *VPD* than to *SWC* [17,18], but whether this pattern applies to agricultural ecosystems remains unverified.

The ecological implications of *WUE* can be further dissected by decomposing it into gross primary productivity to transpiration ratio (*GPP*/*T*) and *T*/*ET* [5,19]. *GPP*/*T* reflects water use efficiency governed by plant physiological regulation (e.g., stomatal behavior and photosynthetic enzyme activity) [20], whereas *T*/*ET* represents the ratio of water consumption allocated to physical and biological processes at the ecosystem scale [21]. For global ecosystems, Wang et al. (2024) proposed that the sensitivity of *WUE* to *SWC* primarily stemmed from *T*/*ET* responses to *SWC*, while its sensitivity to *VPD* was mainly linked to *VPD*-driven changes in *GPP*/*T* [17]. In forest ecosystems, Nie et al. (2021) demonstrated that *WUE* was largely unaffected by *SWC* under low *VPD* conditions, but its sensitivity to *SWC* increased significantly with rising *VPD*, as higher *SWC* under elevated *VPD* led to a rapid decline in *T*/*ET* [15]. However, agricultural ecosystems, particularly maize (a C4 crop with unique traits such as high photosynthetic efficiency and strong transpiration) [22], may fundamentally alter carbon-water coupling mechanisms. Systematic studies on the quantitative responses of *GPP*/*T* and *T*/*ET* to *SWC* and *VPD* in agroecosystems remain scarce.

Under global climate change, the trends of increasing *VPD* and declining *SWC* are pronounced [23,24,25], with projections indicating intensified frequency and severity of extreme drought events [26]. As one of the world’s three most widely cultivated staple crops, maize production stability is critical for global food security [27,28]. Maize is a C4 crop with a distinctive photosynthetic pathway that confers higher intrinsic water use efficiency compared to C3 crops, as its CO_2_-concentrating mechanism minimizes photorespiration and maintains high carboxylation efficiency even under moderate stomatal closure [29]. This physiological trait allows maize to sustain relatively high carbon assimilation rates while reducing transpirational water loss, thereby optimizing the *GPP*/*T* ratio under favorable conditions [30]. However, the C4 advantage may diminish under combined soil and atmospheric drought, as the coordination between stomatal and mesophyll conductance becomes critical for balancing carbon gain and water conservation [31,32]. While recent studies in forest and global ecosystems have revealed divergent roles of *GPP*/*T* and *T*/*ET* in mediating *WUE* responses to environmental stress [15,17], the relative importance of these two pathways in C4-dominated agricultural ecosystems—particularly in rainfed maize systems facing intensified drought risks—remains poorly understood.

Therefore, selecting a representative C4 agricultural region with escalating drought risks is essential for addressing this knowledge gap. Northeast China, a major maize-producing region, faces escalating drought risks driven by climate change [33,34], necessitating a clear understanding of the response mechanisms of the maize ecosystem *WUE* to *SWC* and *VPD* in this area. This study focused on maize croplands in Northeast China, aiming to (1) quantify and compare the relative contributions of *SWC* and *VPD* to *WUE*; and (2) elucidate whether *WUE* variations were attributed to changes in *GPP*/*T* or *T*/*ET*. By decomposing *WUE* into physiological (*GPP*/*T*) and hydrological (*T*/*ET*) components, this study uniquely elucidates the regulatory pathways of soil and atmospheric dryness on carbon-water coupling in C4 agriculture, providing critical insights for sustainable crop management under intensifying climate stress. The findings will advance the understanding of carbon-water interactions in agricultural ecosystems and provide theoretical support for optimizing water management and enhancing climate resilience.

## 2. Results

### 2.1. Ecosystem WUE, GPP, ET, T, E, T/ET, and GPP/T Under Different SWC and VPD Conditions

**Figure 1:**
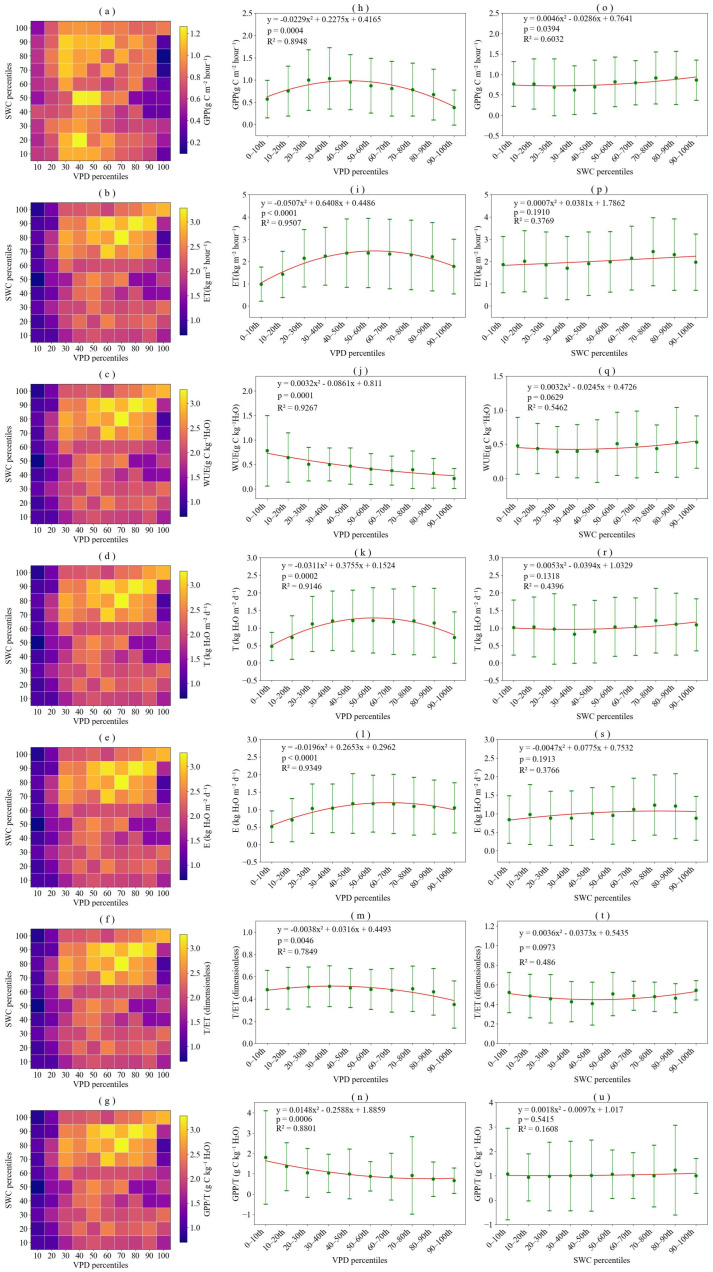
Effects of vapor pressure deficit (*VPD*) and soil water content (*SWC*) on gross primary productivity (*GPP*), evapotranspiration (*ET*), water use efficiency (*WUE*), plant transpiration (*T*), soil evaporation (*E*), transpiration to evapotranspiration ratio (*T*/*ET*), and gross primary productivity to transpiration ratio (*GPP*/*T*) at a half-hourly timescale. (**a**–**g**) Mean values of *GPP*, *ET*, *WUE*, *T*, *E*, *T*/*ET*, and *GPP*/*T* across *VPD* and *SWC* percentile bins; (**h**–**n**) Box plots of *GPP*, *ET*, *WUE*, *T*, *E*, *T*/*ET*, and *GPP*/*T* for each VPD percentile bin; (**o**–**u**) Box plots of *GPP*, *ET*, *WUE*, *T*, *E*, *T*/*ET*, and *GPP*/*T* for each *SWC* percentile bin.

The responses of *WUE*, *GPP*, *ET*, *T*, *E*, *GPP*/*T*, *T*/*ET* and *GPP*/*T* to *VPD* and *SWC* exhibited distinct patterns (Figure 1). Specifically, high *GPP* values were primarily observed under higher *SWC* and moderate *VPD* conditions (Figure 1a,h,o). Under high *VPD* conditions, the strong atmospheric evaporative demand led to stomatal closure to prevent excessive water loss, which inhibited photosynthesis and resulted in a decline in *GPP*. *ET* initially increased and then decreased with increasing *VPD*, while it consistently increased with rising *SWC* (Figure 1b,i,p). This pattern indicates that, under sufficient soil moisture conditions, low *VPD* promotes evapotranspiration, but once *VPD* exceeds a certain threshold, stomatal regulation constrains *ET*. The variation in *WUE* was co-regulated by *GPP* and *ET*, decreasing with increasing *VPD* and exhibiting an initial increase followed by a decrease with rising *SWC* (Figure 1c,j,q). The trend in *T* was similar to that of *ET* (Figure 1d,k,r), primarily because the study period corresponded to the peak growing season, featuring a high leaf area index, which resulted in a relatively small contribution from *E*. *E* increased with *VPD*, subsequently stabilizing at high *VPD* conditions, and increased with *SWC* (Figure 1e,l,s). The *T*/*ET* initially decreased and then increased with *SWC*, and remained relatively high from VPD bin 1 to bin 9, but decreased significantly at *VPD* bin10 (Figure 1f,m,t). This decline was primarily attributed to the substantial reduction in *T* under high *VPD* conditions due to stomatal closure. Furthermore, *GPP*/*T* decreased with increasing *VPD* and increased slightly with increasing *SWC* (Figure 1g,n,u).

### 2.2. Sensitivity of WUE, GPP/T and T/ET to VPD and SWC

**Figure 2:**
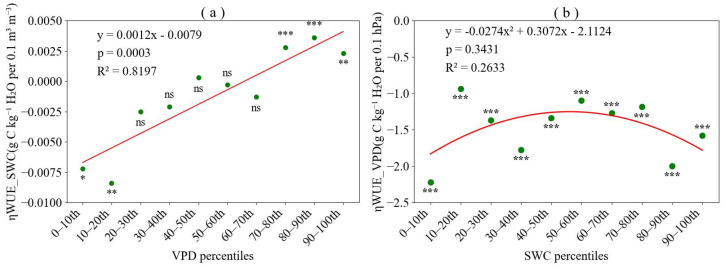
Sensitivity of water use efficiency (*WUE*) to soil water content (*SWC*) and vapor pressure deficit (*VPD*). (**a**) Sensitivity to *SWC* ($\eta_{{\mathrm{WUE}}_{\mathrm{SWC}}}$) across *VPD* percentile bins. (**b**) Sensitivity to *SWC* ($\eta_{{\mathrm{WUE}}_{\mathrm{VPD}}}$) across *SWC* percentile bins. Sensitivity coefficients significantly different from zero are indicated with asterisks (ns = not significant, * *p* < 0.05, ** *p* < 0.01, *** *p* < 0.001).

The sensitivity of *WUE* to *SWC*, denoted as $\eta_{{\mathrm{WUE}}_{\mathrm{SWC}}}$, shifted from negative to positive values with rising *VPD* bins (Figure 2a). This pattern indicated a negative relationship between *WUE* and *SWC* under low *VPD* conditions and a positive relationship under high *VPD* conditions. With rising *VPD*, the negative sensitivity of *WUE* to *SWC* gradually weakened, while the positive sensitivity strengthened. Low and high *VPD* ranges amplified the negative and positive sensitivity of *WUE* to *SWC*, respectively. In contrast, under moderate *SWC* conditions (20th–70th percentiles), the sensitivity of *WUE* to *SWC* approached zero.

Conversely, the sensitivity of *WUE* to *VPD*, represented as $\eta_{{\mathrm{WUE}}_{\mathrm{VPD}}}$, remained consistently negative across all *SWC* bins, reflecting a stable inverse relationship between *WUE* and *VPD* (Figure 2b). Across all ten *SWC* intervals, *WUE* exhibited a significantly negative correlation with *VPD*. The sensitivity of $\eta_{{\mathrm{WUE}}_{\mathrm{VPD}}}$ to increasing *SWC* bins followed a convex pattern—initially weakening and then strengthening. Specifically, both extremely low (0–10th and 10–20th percentiles) and extremely high (80–90th and 90–100th percentiles) SWC conditions enhanced the sensitivity of *WUE* to *VPD*.

**Figure 3:**
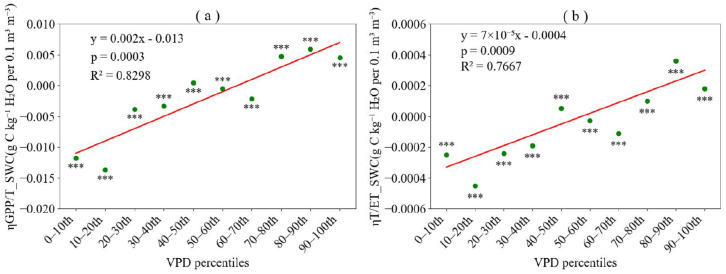
Sensitivity of gross primary productivity to transpiration ratio (*GPP*/*T*) and transpiration to evapotranspiration ratio (*T*/*ET*) to soil water content (*SWC*). (**a**) Sensitivity of *GPP*/*T* to *SWC* ($\eta_{{\mathrm{GPP}/T}_{\mathrm{SWC}}}$) across *VPD* percentile bins. (**b**) Sensitivity of *T*/*ET* to *SWC* ($\eta_{{T/\mathrm{ET}}_{\mathrm{SWC}}}$) across *VPD* percentile bins. Sensitivity coefficients significantly different from zero are indicated with asterisks (*** *p* < 0.001).

As *WUE* can be determined by both *GPP*/*T* and *T*/*ET*, we calculated the sensitivity coefficients of *GPP*/*T* and *T*/*ET* to *SWC* and *VPD*, respectively, to elucidate their respective impacts on *WUE* variations and their responses to *SWC* and *VPD*. The sensitivity of both *GPP*/*T* and *T*/*ET* to *SWC*, denoted as $\eta_{{\mathrm{GPP}/T}_{\mathrm{SWC}}}$ and $\eta_{{T/\mathrm{ET}}_{\mathrm{SWC}}}$, increased markedly from negative to positive values with rising *VPD* bins (Figure 3). Specifically, $\eta_{{\mathrm{GPP}/T}_{\mathrm{SWC}}}$ ranged from −0.0137 to 0.0059, while $\eta_{{T/\mathrm{ET}}_{\mathrm{SWC}}}$ ranged from −0.0005 to 0.0004. The average absolute values of $\eta_{{\mathrm{GPP}/T}_{\mathrm{SWC}}}$ and $\eta_{{T/\mathrm{ET}}_{\mathrm{SWC}}}$ were 0.0051 and 0.0002, respectively, indicating that the sensitivity of *GPP*/*T* to *SWC* had a considerably greater influence on *WUE* than did the sensitivity of *T*/*ET* to *SWC*.

Overall, variations in *SWC* primarily influenced *WUE* by regulating *GPP*/*T*, whereas *T*/*ET* exerted relatively limited direct impacts on *WUE*. The effect of *SWC* on *WUE* differed markedly across *VPD* conditions. Under low *VPD*, increased soil moisture tended to suppress *GPP*/*T*, thereby reducing ecosystem *WUE*. Conversely, under high *VPD*, elevated soil moisture generally promoted *GPP*/*T*, consequently enhancing ecosystem *WUE*. This pattern aligned with the direct effects of *SWC* on *WUE* observed in Figure 2a. These findings further underscore the dominant role of *GPP*/*T* in mediating the regulation of *WUE* by *SWC*, particularly under contrasting high and low *VPD* environments. In comparison, *T*/*ET* exhibited a relatively weak response to changes in *SWC*, suggesting that the responses of ecosystem *WUE* to soil moisture and atmospheric dryness were primarily governed by plant physiological regulation (as reflected in *GPP*/*T*), rather than by the partitioning of water loss (*T*/*ET*).

**Figure 4:**
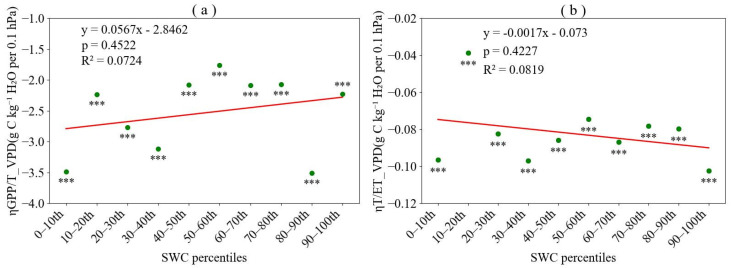
Sensitivity of gross primary productivity to transpiration ratio (*GPP*/*T*) and transpiration to evapotranspiration ratio (*T*/*ET*) to vapor pressure deficit (*VPD*). (**a**) Sensitivity of *GPP*/*T* to *VPD* ($\eta_{{\mathrm{GPP}/T}_{\mathrm{VPD}}}$) across *SWC* percentile bins; (**b**) Sensitivity of *T*/*ET* to *VPD* ($\eta_{{T/\mathrm{ET}}_{\mathrm{VPD}}}$) across *SWC* percentile bins. Sensitivity coefficients significantly different from zero are indicated with asterisks (*** *p* < 0.001).

The sensitivity of both *GPP*/*T* and *T*/*ET* to *VPD* was consistently negative across ten *SWC* bins (Figure 4). Specifically, $\eta_{{\mathrm{GPP}/T}_{\mathrm{VPD}}}$ ranged from −3.5095 to −1.7594 with an average absolute value of 2.5342, while $\eta_{{T/\mathrm{ET}}_{\mathrm{VPD}}}$ ranged from −0.1023 to −0.0387 with an average value of 0.0822. The considerably larger magnitude of $\eta_{{\mathrm{GPP}/T}_{\mathrm{VPD}}}$ indicated that *GPP*/*T* exhibited substantially greater sensitivity to *VPD* than did *T*/*ET*. Furthermore, both sensitivity coefficients showed no significant trends across the SWC gradient, remaining relatively stable as soil moisture bins varied. These results demonstrated that the negative response of ecosystem WUE to increasing VPD was primarily driven by reductions in *GPP*/*T*, reflecting the dominant role of plant physiological regulation under atmospheric dryness, whereas changes in water partitioning (*T*/*ET*) contributed only minimally to VPD-induced *WUE* variations.

### 2.3. Contribution of GPP/T and T/ET to Sensitivity of WUE to VPD and SWC

**Figure 5:**
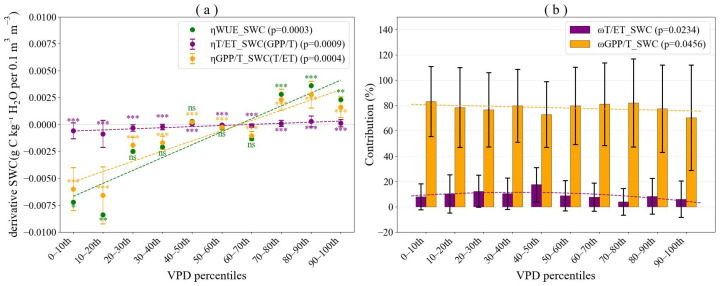
Decomposition of the sensitivity of ecosystem water use efficiency (*WUE*) to soil water content (*SWC*) into contributions from (*GPP*/*T*) and (*T*/*ET*). (**a**) Sensitivity of *WUE* to *SWC* caused by changes in $\eta_{{\mathrm{GPP}/T}_{\mathrm{SWC}}}\times \frac{T}{\mathrm{ET}}$ and $\eta_{{T/\mathrm{ET}}_{\mathrm{SWC}}}\times \frac{\mathrm{GPP}}{T}$. (**b**) Relative contributions of $\eta_{{\mathrm{GPP}/T}_{\mathrm{SWC}}}\times \frac{T}{\mathrm{ET}}$ and $\eta_{{T/\mathrm{ET}}_{\mathrm{SWC}}}\times \frac{\mathrm{GPP}}{T}$ to $\eta_{{\mathrm{WUE}}_{\mathrm{SWC}}}$($\omega_{{T/\mathrm{ET}}_{\mathrm{SWC}}}$ and $\omega_{{\mathrm{GPP}/T}_{\mathrm{SWC}}}$). Here $\eta_{{\mathrm{GPP}/T}_{\mathrm{SWC}}}$, $\eta_{{T/\mathrm{ET}}_{\mathrm{SWC}}}$, and $\eta_{{\mathrm{WUE}}_{\mathrm{SWC}}}$ represent the sensitivity of *GPP*/*T*, *T*/*ET*, and *WUE* to *SWC*, respectively. Sensitivity coefficients significantly different from zero are indicated with asterisks (ns = not significant, * *p* < 0.05, ** *p* < 0.01, *** *p* < 0.001).

According to Equation (7), $\eta_{{\mathrm{WUE}}_{\mathrm{SWC}}}$ is composed of $\eta_{{\mathrm{GPP}/T}_{\mathrm{SWC}}}\times \frac{T}{\mathrm{ET}}$ and $\eta_{{T/\mathrm{ET}}_{\mathrm{SWC}}}\times \frac{\mathrm{GPP}}{T}$. $\eta_{{\mathrm{WUE}}_{\mathrm{SWC}}}$, $\eta_{{\mathrm{GPP}/T}_{\mathrm{SWC}}}\times \frac{T}{\mathrm{ET}}$ and $\eta_{{T/\mathrm{ET}}_{\mathrm{SWC}}}\times \frac{\mathrm{GPP}}{T}$ ranged from −0.0084 to 0.0036, −0.0066 to 0.0028, and −0.0009 to 0.0003, with average values of −0.0013, −0.0011, and −0.0001, respectively (Figure 5a). These results revealed that $\eta_{{\mathrm{GPP}/T}_{\mathrm{SWC}}}\times \frac{T}{\mathrm{ET}}$ consistently dominated over $\eta_{{T/\mathrm{ET}}_{\mathrm{SWC}}}\times \frac{\mathrm{GPP}}{T}$ across all VPD bins. Moreover, all three terms increased significantly with rising *VPD* bins, indicating that the sensitivity of *WUE* to *SWC* strengthened as *VPD* increased, and the absolute contributions of both components also increased accordingly.

Further examination of $\omega_{{\mathrm{GPP}/T}_{\mathrm{SWC}}}$ and $\omega_{{T/\mathrm{ET}}_{\mathrm{SWC}}}$ showed that the relative contribution of *SWC* to *WUE* sensitivity via *GPP*/*T* ranged from 70.25% to 83.30%, whereas that via *T*/*ET* ranged from 3.88% to 17.55% across varying VPD conditions (Figure 5b). These findings clearly demonstrated that the sensitivity of *WUE* to *SWC* was predominantly driven by *GPP*/*T*. Although the contribution of *GPP*/*T* gradually decreased with increasing *VPD*, it remained dominant across all *VPD* bins. In contrast, the contribution of *T*/*ET* first increased and then decreased with rising *VPD*, exhibiting a pattern of initial enhancement followed by suppression. The relative contributions quantitatively confirmed that plant physiological regulation (reflected in *GPP*/*T*), rather than water partitioning (*T*/*ET*), served as the primary mechanism through which soil moisture influences ecosystem water use efficiency under changing atmospheric demand conditions.

**Figure 6:**
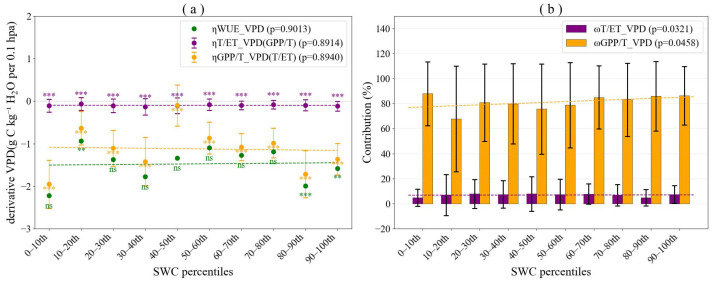
Decomposition of the sensitivity of ecosystem water use efficiency (*WUE*) to vapor pressure deficit (*VPD*) into contributions from (*GPP*/*T*) and (*T*/*ET*). (**a**) Sensitivity of *WUE* to *VPD* caused by changes in $\eta_{{\mathrm{GPP}/T}_{\mathrm{VPD}}}\times \frac{T}{\mathrm{ET}}$ and $\eta_{{T/\mathrm{ET}}_{\mathrm{VPD}}}\times \frac{\mathrm{GPP}}{T}$. (**b**) Relative contributions of $\eta_{{\mathrm{GPP}/T}_{\mathrm{VPD}}}\times \frac{T}{\mathrm{ET}}$ and $\eta_{{T/\mathrm{ET}}_{\mathrm{VPD}}}\times \frac{\mathrm{GPP}}{T}$ to $\eta_{{\mathrm{WUE}}_{\mathrm{VPD}}}$($\omega_{{T/\mathrm{ET}}_{\mathrm{VPD}}}$ and $\omega_{{\mathrm{GPP}/T}_{\mathrm{VPD}}}$). Here $\eta_{{\mathrm{GPP}/T}_{\mathrm{VPD}}}$, $\eta_{{T/\mathrm{ET}}_{\mathrm{VPD}}}$, and $\eta_{{\mathrm{WUE}}_{\mathrm{VPD}}}$ represent the sensitivity of *GPP*/*T*, *T*/*ET*, and *WUE* to *VPD*, respectively. Sensitivity coefficients significantly different from zero are indicated with asterisks (ns = not significant, ** *p* < 0.01, *** *p* < 0.001).

According to Equation (8), $\eta_{{\mathrm{WUE}}_{\mathrm{VPD}}}$ is composed of $\eta_{{\mathrm{GPP}/T}_{\mathrm{VPD}}}\times \frac{T}{\mathrm{ET}}$ and $\eta_{{T/\mathrm{ET}}_{\mathrm{VPD}}}\times \frac{\mathrm{GPP}}{T}$. $\eta_{{\mathrm{WUE}}_{\mathrm{VPD}}}$, $\eta_{{\mathrm{GPP}/T}_{\mathrm{VPD}}}\times \frac{T}{\mathrm{ET}}$ and $\eta_{{T/\mathrm{ET}}_{\mathrm{VPD}}}\times \frac{\mathrm{GPP}}{T}$ ranged from −2.2184 to −0.9367, −1.9513 to −0.1011, and −0.1337 to −0.0665, with average values of −1.4765, −1.1222, and −0.0994, respectively (Figure 6a). These results revealed that the absolute values of $\eta_{{\mathrm{GPP}/T}_{\mathrm{VPD}}}\times \frac{T}{\mathrm{ET}}$ consistently dominated over those of $\eta_{{T/\mathrm{ET}}_{\mathrm{VPD}}}\times \frac{\mathrm{GPP}}{T}$ across all SWC bins. The lack of significant variation in $\eta_{{\mathrm{WUE}}_{\mathrm{VPD}}}$, $\eta_{{\mathrm{GPP}/T}_{\mathrm{VPD}}}\times \frac{T}{\mathrm{ET}}$ and $\eta_{{T/\mathrm{ET}}_{\mathrm{VPD}}}\times \frac{\mathrm{GPP}}{T}$ with increasing *VPD* further suggested that *SWC* exerted a relatively weak influence on the sensitivity of *WUE* to *VPD*. Further examination of $\omega_{{T/\mathrm{ET}}_{\mathrm{VPD}}}$ and $\omega_{{\mathrm{GPP}/T}_{\mathrm{VPD}}}$ revealed that the relative contribution of *VPD* to *WUE* sensitivity via *GPP*/*T* ranged from 67.89% to 87.96%, whereas that via *T*/*ET* ranged from 4.78% to 7.92% across varying soil moisture conditions (Figure 6b). These findings clearly demonstrated that the sensitivity of *WUE* to *VPD* was predominantly driven by *GPP*/*T*.

## 3. Discussion

### 3.1. Ecosystem WUE Exhibits Stronger Sensitivity to VPD than to SWC

Understanding how ecosystem *WUE* responds to *SWC* and *VPD* is critical for predicting carbon-water coupling under climate change [17]. Our results demonstrated that *WUE* in the rainfed maize ecosystem of Northeast China was more sensitive to *VPD* than to *SWC*, consistent with recent findings in global terrestrial ecosystems [17,35]. This heightened sensitivity to atmospheric dryness likely arose from the direct and rapid physiological responses of stomata to changes in atmospheric evaporative demand [23]. Stomata respond almost instantaneously to *VPD* fluctuations to prevent excessive water loss, thereby modulating both carbon uptake (*GPP*) and water loss (*ET*) simultaneously [6]. In contrast, *SWC* influences *WUE* indirectly through gradual changes in soil water availability and plant hydraulic status, which operate on longer time scales [6].

The contrasting patterns of *WUE* sensitivity to *SWC* across *VPD* gradients further illuminated the interactive effects of soil and atmospheric dryness. Under low *VPD* conditions, *WUE* decreased with increasing *SWC* (negative $\eta_{{\mathrm{WUE}}_{\mathrm{SWC}}}$ in Figure 2a), primarily because enhanced soil moisture promoted *ET* more substantially than *GPP* (Figure 1h,i). This aligned with observations in forest ecosystems where high soil moisture under low atmospheric demand led to non-stomatal water loss and reduced *WUE* [15]. Under high *VPD* conditions, however, *WUE* increased with SWC (positive $\eta_{{\mathrm{WUE}}_{\mathrm{SWC}}}$ in Figure 2a), reflecting the critical role of adequate soil moisture in sustaining stomatal conductance and photosynthesis when atmospheric dryness would otherwise induce severe stomatal closure [36]. The near-zero sensitivity under moderate VPD (20th–70th percentiles in Figure 2a) suggested a compensatory effect where the opposing influences of *SWC* on *GPP* and *ET* approximately canceled out. The persistent negative $\eta_{{\mathrm{WUE}}_{\mathrm{VPD}}}$ across all SWC bins underscored the universally suppressive effect of atmospheric dryness on *WUE* (Figure 2b). This finding corroborated global-scale analyses showing that rising *VPD* consistently reduces *WUE* across diverse ecosystems [37]. Notably, both extremely low and high *SWC* amplified the negative sensitivity of *WUE* to *VPD*, suggesting that either water scarcity or waterlogging can exacerbate the detrimental effects of atmospheric dryness. Under low *SWC*, plants lack the hydraulic capacity to meet high evaporative demand, leading to severe stomatal limitation of photosynthesis [9,38,39]. Under high *SWC*, excessive soil moisture may impair root function and reduce photosynthetic capacity, making plants more vulnerable to *VPD*-induced stress [40,41,42].

From an agricultural water management perspective, these findings highlight that mitigating *VPD* stress should be a priority in rainfed maize systems, given its consistently negative impact on *WUE* across all soil moisture conditions. Practices such as optimizing planting density to improve canopy microclimate [43], using mulching to reduce surface temperature [44], or selecting varieties with efficient stomatal regulation [45] could help buffer crops against rising atmospheric demand. Moreover, the context-dependent role of *SWC*, negative under low *VPD* but positive under high *VPD*, suggests that irrigation strategies should be targeted to high *VPD* periods rather than applied uniformly in response to soil moisture deficits alone.

### 3.2. GPP/T Dominates the Regulation of WUE by Both SWC and VPD

Decomposing *WUE* into its physiological (*GPP*/*T*) and hydrological (*T*/*ET*) components revealed distinct regulatory pathways through which *SWC* and *VPD* influence ecosystem carbon-water coupling. Our results unequivocally demonstrated that *GPP*/*T* served as the primary driver of *WUE* sensitivity to both environmental factors, with its contributions exceeding 70.25% to 83.30% for *SWC* sensitivity and 67.89% to 87.96% for VPD sensitivity across all conditions (Figure 5b and Figure 6b). This finding aligned with the framework for global ecosystems, confirming that plant physiological regulation, rather than evapotranspiration partitioning, governs *WUE* responses to environmental stress [17,29,32].

The dominant role of *GPP*/*T* reflects the direct impact of stomatal behavior on the balance between carbon assimilation and water loss at the leaf level [9]. Under varying *SWC* conditions, changes in *GPP*/*T* arise from soil moisture effects on photosynthetic capacity and stomatal conductance [46]. The shift from negative to positive $\eta_{{\mathrm{GPP}/T}_{\mathrm{SWC}}}$ with increasing *VPD* (Figure 3a) indicated that soil moisture limitation became increasingly important for photosynthesis when atmospheric demand was high—a phenomenon consistent with the “compound dry and hot” stress mechanism [26]. Under high *VPD*, adequate *SWC* maintained photosynthetic enzyme activity and electron transport rates, whereas under low *VPD*, excessive *SWC* might actually downregulate photosynthesis through non-stomatal mechanisms [47,48].

For *VPD* effects, the consistently negative $\eta_{{\mathrm{GPP}/T}_{\mathrm{VPD}}}$ across all SWC bins (Figure 4a) reflects the universal suppression of intrinsic water use efficiency by atmospheric dryness. This suppression occurs because stomatal closure in response to high VPD reduces CO_2_ diffusion more severely than it reduces transpiration, particularly in C4 crops like maize that maintain high photosynthetic efficiency under moderate stress [22,49]. The relatively small magnitude of $\eta_{{T/\mathrm{ET}}_{\mathrm{VPD}}}$ compared to $\eta_{{\mathrm{GPP}/T}_{\mathrm{VPD}}}$ (average absolute values 0.0822 vs. 2.5342), indicated that changes in the transpiration fraction contributed minimally to *VPD*-induced *WUE* variations. This suggested that in dense maize canopies during peak growing season, *T*/*ET* remained relatively stable because soil evaporation was largely suppressed by canopy cover [12].

The minimal contribution of *T*/*ET* to *WUE* sensitivity (typically lower than 18% in Figure 5b for *SWC* and lower than 8% for *VPD* in Figure 6b) contrasts with findings in forest ecosystems, where *T*/*ET* played a more substantial role in mediating *SWC* effects on *WUE* [15]. This discrepancy likely reflected fundamental differences in canopy structure between agricultural and forest ecosystems. In maize croplands, the canopy is characterized by a uniform and dense structure with a high leaf area index during the peak growing season (15 May to 15 September), resulting in nearly complete soil coverage that effectively suppresses soil evaporation and buffers *T*/*ET* against environmental fluctuations [50,51]. In contrast, forest ecosystems exhibit vertically stratified canopies with gaps, allowing for more dynamic adjustments in *T*/*ET* in response to soil moisture changes [52]. During early or late growth stages when canopy cover is incomplete, *E* may contribute more substantially to *ET* [53,54], potentially altering the relative importance of *T*/*ET* in mediating *WUE* responses.

**Figure 7:**
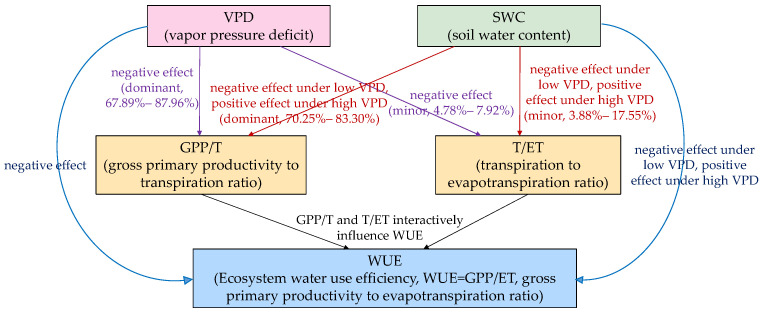
Conceptual diagram of the causal pathways through which soil water content (*SWC*) and vapor pressure deficit (*VPD*) affect ecosystem water use efficiency (*WUE*) in a rainfed maize cropland.

To visually synthesize the causal mechanisms identified in this study, we present a conceptual diagram (Figure 7). The diagram integrates the key findings: *VPD* directly suppresses *WUE* through its negative effect on *GPP*/*T*, whereas *SWC* exhibits a context-dependent influence—beneficial under high *VPD* but detrimental under low *VPD*—also primarily mediated by *GPP*/*T*. In contrast, *T*/*ET* plays a minor role. This graphical representation helps readers understand the dominant regulatory pathway of plant physiological regulation over evapotranspiration partitioning in this C4 agricultural ecosystem.

These findings carry direct implications for agricultural water management. The dominant role of *GPP*/*T* suggests that management practices aimed at enhancing photosynthetic capacity and stomatal regulation, such as selecting drought-tolerant maize varieties [55], optimizing nitrogen fertilization [56], and improving root-zone water availability through conservation tillage or supplemental irrigation [57], are likely to be more effective than interventions focused solely on reducing soil evaporation. Furthermore, the limited contribution of *T*/*ET* during the peak growing season implies that efforts to partition evapotranspiration may have less practical relevance for water management in dense canopies, whereas maintaining plant physiological function should remain the priority. Under future climate scenarios characterized by increasing *VPD* and more frequent droughts [58,59], these strategies—particularly maintaining adequate soil moisture during high-atmospheric-demand periods and prioritizing plant physiological function—will be essential for sustaining carbon assimilation and water use efficiency in rainfed maize production systems. This mechanistic understanding also advances our ability to model crop responses to climate change, indicating that incorporating plant physiological responses may be more critical than accurately representing evapotranspiration partitioning when predicting WUE dynamics in maize croplands.

### 3.3. Limitations and Future Directions

Several limitations of this study should be acknowledged. First, the maize variety was not specified in the flux dataset, as the observations were originally designed for long-term ecosystem-scale carbon and water flux monitoring rather than variety-specific comparisons. Different maize cultivars may exhibit variation in water use efficiency and stomatal regulation [29], which could influence the generalizability of our findings. Second, although we have described the general agronomic practices such as planting density, no-till, and fertilization, the field management represents a single set of conditions. The observed relationships between *WUE*, *SWC*, and *VPD* may vary under different planting patterns or management intensities [43]. Third, soil physical and chemical properties beyond pH and organic matter content, such as bulk density and hydraulic conductivity, were not available. This may affect root-zone water availability and plant water uptake dynamics [38]. Fourth, our observations were limited to the peak growing season (15 May to 15 September) when canopy cover is complete. The relative importance of *GPP*/*T* and *T*/*ET* in regulating *WUE* may differ during early or late growth stages when canopy cover is incomplete [53,54]. Fifth, the findings are based on a single rainfed maize site in Northeast China with a specific temperate monsoon climate. The observed responses of *WUE* to *SWC* and *VPD* may not be directly transferable to regions with different climatic regimes, such as subtropical or semi-arid zones [60,61], or to irrigated maize systems where water availability is less constrained by precipitation patterns [62,63]. Validation at multiple sites with diverse climatic conditions and management practices is needed to assess the broader applicability of our conclusions.

Future research should incorporate variety-level information, consider a wider range of agronomic practices, extend observations across the full growing season, and include multi-site comparisons across different climatic zones and irrigation regimes to further validate the observed mechanisms.

## 4. Materials and Methods

### 4.1. Flux Data

This study was conducted at the Jinzhou Agricultural Ecosystem Field Experiment Site (41°08′53″ N, 121°12′06″ E, 23 m a.s.l.), a rainfed spring maize cropland in Northeast China. The experimental field and its surrounding areas are flat and open. The region experiences a temperate monsoon climate, with warm, humid summers and dry, cold winters. Long-term records indicate a mean annual air temperature of 10.1 °C and a mean annual precipitation of 580.0 mm, of which nearly 70% falls between June and August. The frost-free period is approximately 180 days. The rainfall regime is dominated by the East Asian summer monsoon, which is the primary driver of summer precipitation in Northeast China. Soils are characterized by a pH of approximately 6.3 and organic matter content ranging from 0.6% to 0.9%. Maize is sown in early May and harvested in late September, with a planting density of approximately 5.1 plants m^−2^. No-till or minimal tillage is employed, and fertilization is applied as NH_4_HCO_3_ at a rate of 300 kg ha^−1^ yr^−1^ at the pre-seeding stage.

Half-hourly flux, meteorological data, and soil water data in 2005–2014 were obtained from ChinaFLUX (http://www.chinaflux.org, 1 July 2025). The eddy covariance system comprised a three-dimensional sonic anemometer (CSAT3, Campbell Scientific, Logan, UT, USA) and an open-path infrared gas analyzer (LI-7500, LI-COR Biosciences, Bourne, MA, USA), installed at 2.5 m above ground. Raw data were sampled at 10 Hz and processed to half-hourly fluxes following standard procedures. Gross primary productivity (*GPP*, mg CO_2_ m^−2^ s^−1^, converted to g C m^−2^ h^−1^) was derived from net ecosystem exchange partitioning, and latent heat flux (*LE*, W m^−2^) was directly measured. Supporting meteorological variables, including air temperature (*T*_a_, °C) and relative humidity (*RH*, %), were recorded using a temperature and humidity probe (HMP45C, Vaisala, Vantaa, Finland) at the same height. Soil water content (*SWC*, m^3^ m^−3^) was monitored at 10 cm depth using time domain reflectometry (CS616, Campbell Scientific, USA). Net radiation and precipitation were measured, respectively, with a four-component net radiometer (CNR1, Kipp & Zonen, Delft, The Netherlands) and a tipping-bucket rain gauge (TE525MM, Campbell Scientific, USA). All flux data underwent coordinate rotation, Webb–Pearman–Leuning (WPL) correction, and canopy storage correction, with quality controlled through the ChinaFLUX technical system [64]. 

To obtain high-quality data, these data needed to meet the following criteria: (1) Exclude data where the half-hourly precipitation exceeds 1 mm, along with data from the subsequent 48 h period. (2) Only consider data with higher net radiation than 50 W m^−2^ during the active growing season from 15 May to 15 September.

During the 2005–2014 growing seasons (15 May to 15 September), the *SWC* and *VPD* ranged from 0.0165 m^3^ m^−3^ to 0.4955 m^3^ m^−3^ and 0.024 kPa to 0.636 kPa, with the mean *SWC* and *VPD* of 0.2707 ± 0.0870 m^3^ m^−3^ (mean ± standard deviation) and 0.260 ± 0.146 kPa.

*ET* (kg m^−2^ h^−1^) was calculated using *LE* as follows [65]

(1) $$\mathrm{ET}=\frac{3600\;\times \;\mathrm{LE}}{\lambda \rho_{w}}$$

where *λ* (MJ kg^−1^) is the latent heat of vaporization of water, *λ* = 2.501 − 0.002361 *T*_a_, *ρ*_w_ (1000 kg m^−3^) is the water density, and 3600 is a time conversion from “s” to “h”.

*WUE* (g C kg^−1^ H_2_O) was determined by dividing *GPP* by *ET*, representing the efficiency of converting water consumed into ecosystem organic material during photosynthesis [50].

(2) $$\mathrm{WUE}=\frac{\mathrm{GPP}}{\mathrm{ET}}$$

### 4.2. Decoupling of SWC and VPD

Land–atmosphere interactions facilitate strong coupling between *SWC* and *VPD* [26]. However, empirical evidence suggests this coupling diminishes at shorter time scales [15,66,67]. Therefore, we applied a decoupling strategy to the half-hourly data. The data were grouped into 10 distinct bins based on the 0–100th percentile of measured *SWC* values: 0–10, 10–20, 20–30, 30–40, 40–50, 50–60, 60–70, 70–80, 80–90, and 90–100 percentile ranges. The same process was applied to the estimated *VPD* data by $\;\mathrm{VPD}\;=\;0.6108\left(1\;-\;\frac{\mathrm{RH}}{100}\right)\exp(\frac{17.27T_{a}}{273.3\;+\;T_{a}})$ [68], generating corresponding bins. The results revealed a weaker correlation between *SWC* and *VPD* at the half-hourly time scale, with a Pearson’s correlation coefficient of −0.076 ± 0.190. The binning method further reduced the correlation between *SWC* and *VPD*, yielding a correlation coefficient of −0.033 ± 0.082 across the bins. As these values approached zero, the post-binning half-hourly SWC and VPD data were successfully decoupled, rendering the two variables independent.

### 4.3. Sensitivity of WUE to SWC and VPD

To independently estimate the sensitivity of *WUE* to *VPD* and *SWC*, we constructed linear regression models between *WUE* and *SWC* in *VPD* bins, as well as between *WUE* and *VPD* in *SWC* bins, respectively, using split-box data.

(3) $$\mathrm{WUE}=\eta_{{\mathrm{WUE}}_{\mathrm{SWC}}^{i}}\;\times \;\mathrm{SWC}+b_{\mathrm{SWCi}}$$

(4) $$\mathrm{WUE}=\eta_{{\mathrm{WUE}}_{\mathrm{VPD}}^{i}}\;\times \;\mathrm{VPD}+b_{\mathrm{VPDi}}$$

where $\eta_{{\mathrm{WUE}}_{\mathrm{SWC}}^{i}}$ indicates the change in *WUE* caused by each 0.1 m^3^⋅m ^−3^ change in *SWC*, reflecting the sensitivity of *WUE* to soil moisture, $\eta_{{\mathrm{WUE}}_{\mathrm{VPD}}^{i}}$ denotes the sensitivity of *WUE* to *VPD*, *b*_SWCi_ and *b*_VPDi_ denote the intercept of the linear models.

All linear regressions (Equations (3) and (4)) were fitted using ordinary least squares. For each regression, the significance of the slope (sensitivity coefficient) was tested using a two-tailed *t*-test against the null hypothesis of zero slope. A *p*-value < 0.05 was considered statistically significant. The number of half-hourly data points (n) in each *VPD* or *SWC* bin was 1403.

### 4.4. Decomposition of WUE

Ecosystem *WUE* is defined as the ratio of *GPP* to *ET* from the entire ecosystem. Since *ET* comprises two ecological processes (soil evaporation *E* and plant transpiration *T*), *WUE* can also be expressed as the product of *GPP*/*T* and *T*/*ET* [15,35].

(5) $$\mathrm{WUE}=\frac{\mathrm{GPP}}{\mathrm{ET}}=\frac{\mathrm{GPP}}{T}\;\times \;\frac{T}{\mathrm{ET}}$$

where *GPP*/*T* refers to the amount of carbon accumulated per unit of water consumed by the plant, and *T*/*ET* describes the relative contribution of water vapor pathways in the ecosystem water cycle, encompassing biotic and abiotic processes.

*T*/*ET* was estimated as the ratio of the apparent to the potential underlying water-use efficiency using half-hourly flux data [69].

(6) $$\frac{T}{\mathrm{ET}}=\frac{u{\mathrm{WUE}}_{a}}{u{\mathrm{WUE}}_{p}}$$

where u*WUE*_a_ is the apparent underlying water-use efficiency, estimated using the linear regression slope between *GPP* × *VPD*^0.5^ and *ET* from an 8-day moving window. u*WUE*_p_ is the potential underlying water-use efficiency, calculated using the 95th percentile regression between half-hourly *GPP* × *VPD*^0.5^ and *ET* during the active growing season.

### 4.5. Evaluating the Sensitivity of GPP/T and T/ET to VPD and SWC

To understand the impact of *SWC* and *VPD* changes on *WUE*, we explored the sensitivity of *GPP*/*T* and *T*/*ET* to *VPD* and *SWC*, respectively. The full derivative of *WUE* with respect to *SWC* and *VPD* [15,36] can be expressed as

(7) $$\eta_{{\mathrm{WUE}}_{\mathrm{SWC}}}=\frac{d\mathrm{WUE}}{d\mathrm{SWC}}=\frac{d(\frac{\mathrm{GPP}}{\mathrm{ET}})}{d\mathrm{SWC}}=\frac{\partial (\frac{\mathrm{GPP}}{T})}{\partial \mathrm{SWC}}\;\times \;\frac{T}{\mathrm{ET}}+\frac{\partial (\frac{T}{\mathrm{ET}})}{\partial \mathrm{SWC}}\;\times \;\frac{\mathrm{GPP}}{T}=\eta_{{\mathrm{GPP}/T}_{\mathrm{SWC}}}\;\times \;\frac{T}{\mathrm{ET}}+\eta_{{T/\mathrm{ET}}_{\mathrm{SWC}}}\;\times \;\mathrm{WUE}\;÷\;\frac{T}{\mathrm{ET}}$$

(8) $$\eta_{{\mathrm{WUE}}_{\mathrm{VPD}}}=\frac{d\mathrm{WUE}}{d\mathrm{VPD}}=\frac{d(\frac{\mathrm{GPP}}{\mathrm{ET}})}{d\mathrm{VPD}}=\frac{\partial (\frac{\mathrm{GPP}}{T})}{\partial \mathrm{VPD}}\;\times \;\frac{T}{\mathrm{ET}}+\frac{\partial (\frac{T}{\mathrm{ET}})}{\partial \mathrm{VPD}}\;\times \;\frac{\mathrm{GPP}}{T}=\eta_{{\mathrm{GPP}/T}_{\mathrm{VPD}}}\;\times \;\frac{T}{\mathrm{ET}}+\eta_{{T/\mathrm{ET}}_{\mathrm{VPD}}}\;\times \;\mathrm{WUE}\;÷\;\frac{T}{\mathrm{ET}}$$

where $\eta_{{\mathrm{GPP}/T}_{\mathrm{SWC}}}=\frac{\partial (\frac{\mathrm{GPP}}{T})}{\partial \mathrm{SWC}}$ and $\eta_{{T/\mathrm{ET}}_{\mathrm{SWC}}}=\frac{\partial (\frac{T}{\mathrm{ET}})}{\partial \mathrm{SWC}}$ are, respectively, the sensitivities of *WUE* to *SWC* based on variations in *GPP*/*T* and *T*/*ET*, and $\eta_{{\mathrm{GPP}/T}_{\mathrm{VPD}}}=\frac{\partial (\frac{\mathrm{GPP}}{T})}{\partial \mathrm{VPD}}$ and $\eta_{{T/\mathrm{ET}}_{\mathrm{VPD}}}=\frac{\partial (\frac{T}{\mathrm{ET}})}{\partial \mathrm{VPD}}$ are, respectively, the sensitivities of *WUE* to *VPD* based on variations in *GPP*/*T* and *T*/*ET*. Thus,

(9) $$\;\mathrm{WUE}=-\frac{\eta_{{\mathrm{GPP}/T}_{\mathrm{SWC}}}}{\eta_{{T/\mathrm{ET}}_{\mathrm{SWC}}}}\;\times \;{\left(\frac{T}{\mathrm{ET}}\right)}^{2}+\frac{\eta_{{\mathrm{WUE}}_{\mathrm{SWC}}}}{\eta_{{T/\mathrm{ET}}_{\mathrm{SWC}}}}\;\times \;\frac{T}{\mathrm{ET}}$$

(10) $$\;\mathrm{WUE}=-\frac{\eta_{{\mathrm{GPP}/T}_{\mathrm{VPD}}}}{\eta_{{T/\mathrm{ET}}_{\mathrm{VPD}}}}\;\times \;{\left(\frac{T}{\mathrm{ET}}\right)}^{2}+\frac{\eta_{{\mathrm{WUE}}_{\mathrm{VPD}}}}{\eta_{{T/\mathrm{ET}}_{\mathrm{VPD}}}^{*}}\;\times \;\frac{T}{\mathrm{ET}}$$

In Equations (9) and (10), *WUE*, $\eta_{{\mathrm{WUE}}_{\mathrm{SWC}}}$, $\eta_{{\mathrm{WUE}}_{\mathrm{VPD}}}$ and *T/ET* were calculated, respectively, from Equations (2)–(4) and (6). $\eta_{{\mathrm{GPP}/T}_{\mathrm{SWC}}}$, $\eta_{{T/\mathrm{ET}}_{\mathrm{SWC}}}$ in Equation (9) and $\eta_{{\mathrm{GPP}/T}_{\mathrm{VPD}}}$, $\eta_{{T/\mathrm{ET}}_{\mathrm{VPD}}}$ were fitted by nonlinear regression based on an interactive method, minimizing the mean squared residuals in each *VPD* bin and *SWC* bin, respectively.

Nonlinear regressions (Equations (9) and (10)) were performed using the Levenberg–Marquardt algorithm to minimize the mean squared residuals. The significance of the fitted parameters was assessed by *t*-tests, and *p* < 0.05 was considered significant.

The sensitivity of *WUE* on *SWC* can be divided into $\frac{\partial (\frac{\mathrm{GPP}}{T})}{\partial \mathrm{SWC}}\times \frac{T}{\mathrm{ET}}$ and $\frac{\partial (\frac{T}{\mathrm{ET}})}{\partial \mathrm{SWC}}\times \frac{\mathrm{GPP}}{T}$ based on Equation (7), and their contributions to *η*_WUE_ can be calculated as

(11) $$\omega_{{\mathrm{GPP}/T}_{\mathrm{SWC}}}=100\%\;\times \;\frac{\;\frac{\partial (\frac{\mathrm{GPP}}{T})}{\partial \mathrm{SWC}}\;\times \;\frac{T}{\mathrm{ET}}}{\eta_{{\mathrm{WUE}}_{\mathrm{SWC}}}}=100\%\;\times \;\frac{\eta_{{\mathrm{GPP}/T}_{\mathrm{SWC}}}\;\times \;\frac{T}{\mathrm{ET}}}{\eta_{{\mathrm{WUE}}_{\mathrm{SWC}}}}$$

(12) $$\omega_{{T/\mathrm{ET}}_{\mathrm{SWC}}}=100\%\;\times \;\frac{\frac{\partial (\frac{T}{\mathrm{ET}})}{\partial \mathrm{SWC}}\;\times \;\frac{\mathrm{GPP}}{T}}{\eta_{{\mathrm{WUE}}_{\mathrm{SWC}}}}=100\%\;\times \;\frac{\eta_{{T/\mathrm{ET}}_{\mathrm{SWC}}}\;\times \;\frac{\mathrm{GPP}}{T}}{\eta_{{\mathrm{WUE}}_{\mathrm{SWC}}}}$$

where $\omega_{{\mathrm{GPP}/T}_{\mathrm{SWC}}}$ and $\omega_{{T/\mathrm{ET}}_{\mathrm{SWC}}}$ denote, respectively, the contribution of $\frac{\partial (\frac{\mathrm{GPP}}{T})}{\partial \mathrm{SWC}}\times \frac{T}{\mathrm{ET}}$ and $\frac{\partial (\frac{T}{\mathrm{ET}})}{\partial \mathrm{SWC}}\times \frac{\mathrm{GPP}}{T}$ to $\eta_{{\mathrm{WUE}}_{\mathrm{SWC}}}$.

The sensitivity of *WUE* to *VPD* can be divided into $\frac{\partial (\frac{\mathrm{GPP}}{T})}{\partial \mathrm{VPD}}\times \frac{T}{\mathrm{ET}}$ and $\frac{\partial (\frac{T}{\mathrm{ET}})}{\partial \mathrm{VPD}}\times \frac{\mathrm{GPP}}{T}$ based on Equation (8), and their contributions to *η*_WUE_ can be calculated as

(13) $$\omega_{{\mathrm{GPP}/T}_{\mathrm{VPD}}}=100\%\;\times \;\frac{\;\frac{\partial (\frac{\mathrm{GPP}}{T})}{\partial \mathrm{VPD}}\;\times \;\frac{T}{\mathrm{ET}}}{\eta_{{\mathrm{WUE}}_{\mathrm{VPD}}}}=100\%\;\times \;\frac{\eta_{{\mathrm{GPP}/T}_{\mathrm{VPD}}}\;\times \;\frac{T}{\mathrm{ET}}}{\eta_{{\mathrm{WUE}}_{\mathrm{VPD}}}}$$

(14) $$\omega_{{T/\mathrm{ET}}_{\mathrm{VPD}}}=100\%\;\times \;\frac{\frac{\partial (\frac{T}{\mathrm{ET}})}{\partial \mathrm{VPD}}\;\times \;\frac{\mathrm{GPP}}{T}}{\eta_{{\mathrm{WUE}}_{\mathrm{VPD}}}}=100\%\;\times \;\frac{\eta_{{T/\mathrm{ET}}_{\mathrm{VPD}}}\;\times \;\frac{\mathrm{GPP}}{T}}{\eta_{{\mathrm{WUE}}_{\mathrm{VPD}}}}$$

where $\omega_{{\mathrm{GPP}/T}_{\mathrm{VPD}}}$ and $\omega_{{T/\mathrm{ET}}_{\mathrm{VPD}}}$ denote, respectively, the contribution of $\frac{\partial (\frac{\mathrm{GPP}}{T})}{\partial \mathrm{VPD}}\times \frac{T}{\mathrm{ET}}$ and $\frac{\partial (\frac{T}{\mathrm{ET}})}{\partial \mathrm{VPD}}\times \frac{\mathrm{GPP}}{T}$ to $\eta_{{\mathrm{WUE}}_{\mathrm{VPD}}}$.

## 5. Conclusions

This study investigated the responses of ecosystem water use efficiency (*WUE*) to soil water content (*SWC*) and vapor pressure deficit (*VPD*) in a rainfed maize cropland in Northeast China using long-term eddy covariance flux data. By decomposing *WUE* into gross primary productivity to transpiration ratio (*GPP*/*T*) and Transpiration to evapotranspiration ratio (*T*/*ET*), we elucidated the distinct pathways through which soil moisture and atmospheric dryness regulate carbon-water coupling in this agricultural ecosystem.

Results demonstrated that *WUE* exhibited stronger sensitivity to *VPD* than to *SWC*, with consistently negative responses to atmospheric dryness across all soil moisture conditions. The sensitivity of *WUE* to *SWC* shifted from negative to positive with increasing *VPD*. Decomposition analysis revealed that *GPP*/*T* dominated the regulation of *WUE* by both environmental factors, contributing 70.25–83.30% to *SWC* sensitivity and 67.89–87.96% to *VPD* sensitivity across all conditions, while *T*/*ET* played a minor role (3.88–17.55% for *SWC*, 4.78–7.92% for *VPD*). The sensitivity of *WUE* to *SWC* was primarily driven by the responsiveness of *GPP*/*T* to soil moisture, particularly under high *VPD* conditions, and the sensitivity to *VPD* was primarily driven by the direct suppression of *GPP*/*T* by atmospheric dryness.

These findings advance the mechanistic understanding of carbon-water interactions in agricultural ecosystems and provide theoretical support for optimizing water management under climate change. From a sustainability perspective, enhancing plant physiological regulation (*GPP*/*T*) rather than solely reducing soil evaporation offers a more effective strategy to improve *WUE* under rising atmospheric dryness, with direct implications for rainfed maize systems in climate-vulnerable regions worldwide. Future research should validate the mechanistic findings of this study over longer time scales and at multiple sites to assess their generalizability across different climatic conditions and agricultural management practices.

## Data Availability

Data will be made available on request.
